# What Has Been the Focus of Sugarcane Research? A Bibliometric Overview

**DOI:** 10.3390/ijerph16183326

**Published:** 2019-09-10

**Authors:** Katia A. Figueroa-Rodríguez, Francisco Hernández-Rosas, Benjamín Figueroa-Sandoval, Joel Velasco-Velasco, Noé Aguilar Rivera

**Affiliations:** 1 Colegio de Postgraduados-Campus Córdoba, Programa de Innovación Agroalimentaria Sustentable, Km. 348 Carretera Córdoba-Veracruz, Congregación Manuel León, Amatlán de los Reyes, Veracruz, CP 94953, Mexico; 2 Colegio de Postgraduados-Campus San Luis, Programa de Innovación en el Manejo de Recursos Naturales, Calle de Iturbide 73, Salinas de Hidalgo, San Luis Potosí, CP 78622, Mexico; 3 Facultad de Ciencias Biológicas y Agropecuarias, Universidad Veracruzana, Peñuela, Amatlán de los Reyes, Veracruz, CP 94945 Mexico

**Keywords:** sugarcane bagasse, bioenergy, ethanol, yield, environmental issues, Saccharum officinarum

## Abstract

Sugarcane is one of the main crops worldwide, and it has an important impact on environmental issues. A bibliometric mapping analysis of the research on sugarcane was carried out, using data on the titles, abstracts, and keywords of articles published in leading journals and other peer-reviewed documents available in the SCOPUS database from 1858 to 2019 (27 August), and this was subsequently analyzed with the software VOSviewer. The three most important countries that publish research and were most-cited regarding sugarcane were Brazil, the USA, and India. The analysis of the co-occurrence of terms shows that the main research areas were sugarcane bagasse and terms related to bioenergy, and on a second level of relevance agronomy topics related to increasing crop yields. This first attempt to visualize the abundance of publications regarding sugarcane in their totality is in itself a good starting point for further scientific discussion.

## 1. Introduction

Sugarcane is one of the most important crops in the world. In 2016, a total of 26,774,304 ha were harvested with 1.93% of the world’s harvested area, which places it as the 12th most important crop globally. For the same year, sugarcane production was 1,890,661,751 tons, placing it as the most important crop in the world in terms of volume and representing 21.1% of the total world crop production. The countries with the largest production volume in 2017 were: Brazil (41% of world production), India (16%), China (6%), and Thailand (6%). The remainder was produced by 100 countries [1]. Sugarcane produces essential products such as sugar, ethanol, and bagasse or lignocellulose [2]. One of the main concerns regarding this crop is its environmental impact [3].

The literature regarding sugarcane is abundant. Most of the previous reviews regarding this crop focus on products [4], or byproducts such as ethanol [5]; many of the publications are not specific to sugarcane, i.e., they focus on comparing sugarcane with other crops or products [6,7]. In other reviews, the process [8,9], its applications [10], and its implications [11] are discussed. Another important topic for reviews is sustainability [12], such as the Life Cycle Assessment (LCA) methodology [9] or waste/residues management [6].

Due to the amount of scientific literature regarding sugarcane research, a data driven approach known as bibliometric mapping, which relies on computer algorithms and visualization techniques, was chosen [13]. The main results are visual representations of the field created with VOSviewer software for bibliometric mapping, showing the relationship among key terms, authors, and countries; data is obtained from the title, abstract, and keywords of scientific publications [14]. These relationships are distributed using a clustering algorithm, allowing us to observe meaningful groups when analyzing the literature [15]. Previous research using scientometric analyses regarding sugarcane does exist, but the foci of those studies differ from the present study, e.g., issues governing the sugarcane supply and processing chain [16], reduction of the scope to a specific scientific area such as chemistry [17], a focus on one country’s case [18], or an analysis of a different time period (1948–1987) [19].

Using bibliometric mapping, an analysis of sugarcane research published between 1858 and 2019 (27 August) was carried out. The uniqueness of the review is in its coverage on a global scale; it considers the main terms researchers have focused on, identifies the most relevant journals and the publications with the highest Impact Factor, and makes comparisons across contributing countries and authors. Thus, the aim of this study was to create a historical landscape of the sugarcane literature on a global level. The results may serve as a means for identifying potential knowledge gaps regarding this crop. The paper is organized as follows; first we present the methodology used in order to perform the bibliometric analysis and mapping. The results section follows, divided into performance and citation analysis as well as scientific mapping. The third section is the discussion of the main terms and finally the main findings are presented in the conclusions section.

## 2. Materials and Methods

The data used in this paper were obtained from the Scopus search engine. Scopus was chosen due to its various advantages over other databases, in particular the superior number of journals [20] and the fact that multidisciplinary databases outperform specialized databases [21]. While Google Scholar is a more comprehensive academic search engine [22], not all of the abstracts are available for analysis. Another reason was the existence of previous studies that used different datasets, such as patents [23], WoS [17], and the CAB Direct online database [18]. The data were obtained using sugarcane or “sugar cane” due to its coverage and to SCOPUS’s lemmatization search properties. It is common for authors who employ scientific names to also include the common name in the abstract; therefore, “*Saccharum officinarum*” was not considered as a keyword. No side functions in Scopus were used, such as time limitations, source type, data, or subject, and the keywords *sugarcane* or “sugar cane” could be present in the article title, abstract, or keywords. Data analysis was performed using the analyze function in the Scopus menu bar. Data was organized by country, subject, document type, affiliation, author, source, and year. The citation analysis was carried out with information also obtained from Scopus, such as number of citations, and top cited articles. No self-citations were excluded.

Normally, in bibliometric studies, not all data can be presented, so this type of study focuses on the most productive countries, authors, institutions, and journals. Previous research used as a cutoff point 100 publications and the top 10 countries [24], while others used the top 30 publications, countries, journals, and institutes [25]. We decided to retain ranking and the top 10, as the cutoff point, as the cutoff point of 100 was too low to discriminate and the top 30 did not allow for easy comprehension of the main trends.

Some graphs were created using SPSS (Statistical Package for Social Science) version 20.0 software for Windows (SPSS Inc. Chicago, Illinois); this software was also used for descriptive statistics (mean ± standard deviation (SD)). Maps were created with Infogram (https://infogram.com).

### Analysis Content

VOSviewer [26] was used to analyze for each year, the title and abstract fields of the included publications. One term map was produced to illustrate a network of recurring keywords. This map shows the co-occurrence of topics and the relative citation impacts. For the term map, only terms that co-occurred at least five times under binary counting were considered; general noun phrases are removed by the software [14]. Of the remaining terms, 3523 met the threshold, with the highest relevance score calculated by VOSviewer. In total, 500 keywords were used to create a term map allowing network visualization. Other maps using the same software were created with co-authorship for countries and for authors; the first had 609 countries. The number of documents per country was 25. 74 countries met the thresholds and all were retained for the map (we eliminated USA as the United States was already considered), for the citation map the rule were at least 3 citations and 25 documents per country. For the authors’ map, there were a total of 63,521 authors; the selection parameters were number of documents per author of 25 and number of citations per author of 10. 288 authors met the thresholds, and 264 authors were selected for the map. For all cases, the following parameters for VOSviewer were used: Cluster minimum of 1, terms ≥ 10, association strength method, visualization scale of 1.39, TLS weight, size of label variation 50%, and line size variation of 24%. Larger bubbles mean that those terms occurred more frequently; irrelevant terms were removed [27].

## 3. Results

In total, there were 31,049 documents concerning sugarcane/sugar cane from 1858 to 2019 (27 August). With regard to document type, 81.5% were articles, 9.9% conference papers, 3.1% reviews, and the rest were book chapters (855), notes (226), conference reviews (102), books (88), letters (84), short surveys (73), errata (66), business articles (63), editorials (55), data papers (8), and an abstract report (1). These documents were downloaded on 27 August 2019, and used in order to analyze publication performance and science mapping.

### 3.1. Performance Analysis

**Figure 1:**
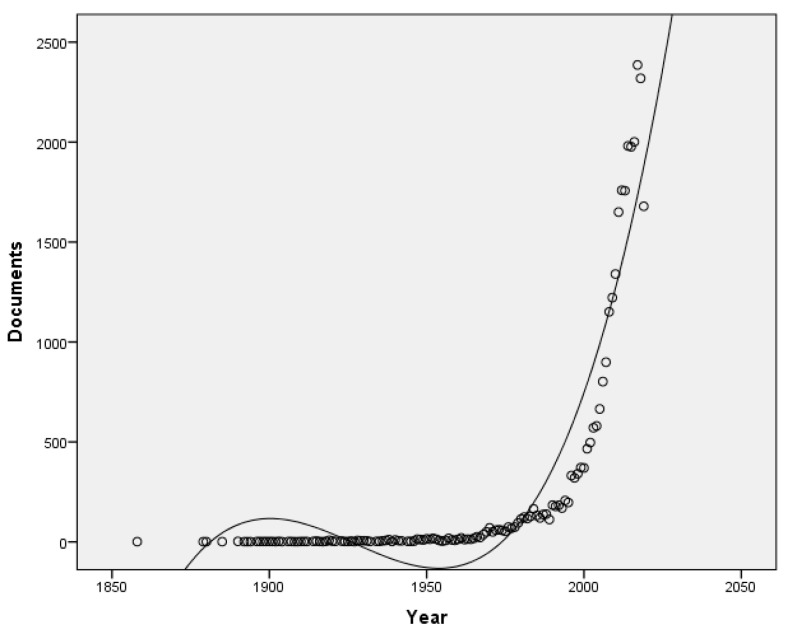
Annual growth of publications from 1858 to 2019 (27 August), Cubic *R*^2^ = 0.877.

The distribution of the publications is presented in Figure 1. The number of publications regarding sugarcane starts in 1858 with an article entitled *A detailed account of experiments and observations upon the sorghum saccharatum or Chinese sugar cane, made with the view of determining its value as a sugar producing plant, from 28 September to 20 December 1857, at Oakhill, Philadelphia county, Pennsylvania*, published in the *Journal of the Franklin Institute* [28]. The next document discusses experiments with fertilizers on sugarcane [29]. The number of publications has been increasing over time, yet 64.6% of the documents were published in the last ten years. The most productive year was 2017, with a total of 2386 documents.

Of the total number of documents, 82.8% have been cited, with an average of 18.40 ± 46.06 citations for the entire period. The maximum number of citations per document is 2271; eight documents had more than 1000, 134 had between 200 and 999 per document, and 3233 articles had been cited once.

Table 1 presents the top ten journals, institutes, and countries that publish scientific research regarding sugarcane. A total of 147 sources exist; the journal with the largest number of publications is Sugar Tech, and the articles from this journal had been cited 4056 times with an average of 5.99 ± 6.80 citations per publication. The journal with the next-largest number of publications was the *International Sugar Journal*, and the documents from this journal had been cited 1443 times with an average of 4.60 ± 5.70 citations per publication. In third place was Bioresource Technology, with a total of 26,017 citations and an average of 49.37 ± 94.41 citations per publication. The main subjects of the journals that published sugarcane topics were: agricultural and biological sciences (29%), environmental sciences (11%), biochemistry, genetics and molecular biology (10%) and engineering (8%). A total of 160 institutes had publications in the SCOPUS database. The most productive institute was the Universidade de Sao Paulo-USP with 2420 publications. Of the top ten institutes, six of them are Brazilian, two are North American, and two Australian.

**Figure 2:**
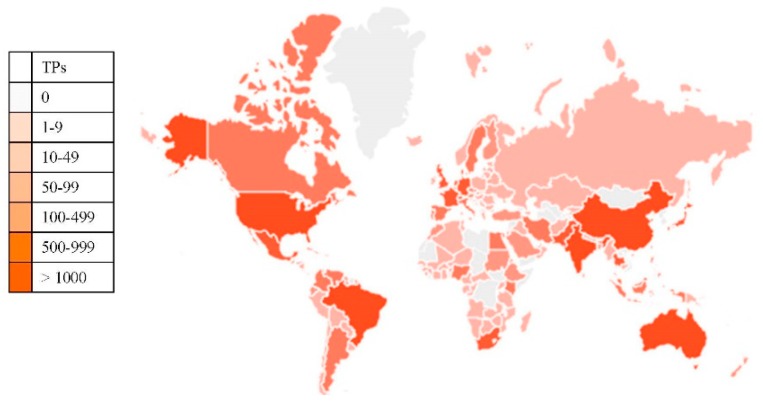
Global distribution of publications related to sugarcane research. TPs: Total Publications. Source: SCOPUS (27 August 2019).

Regarding the authored publications by country, 159 countries were listed, but only 37 countries had more than 100 publications. The country with the largest number of authored publications was Brazil, with 27.2% of the global publications, followed by the United States with 13.5% of the total publications, and India with 13.2% of the total number of authored publications (Figure 2).

### 3.2. Citation Analysis

The top 10 highly-cited papers (see Table 2) are not only focused on sugarcane. For example, Brennan and Owende [30], which is the most-cited article, and Chisti [31] only cite sugarcane to briefly discuss the disadvantages of using this crop to generate biofuels compared to microalgae-based biofuels. The second-most-cited paper presents the genome of a grass related to sugarcane [32], while the articles that focus on sugarcane are oriented towards alternative uses of sugarcane products [33], for example byproducts such as bagasse hemicellulose [34], especially for developing second-generation biofuels produced from non-food biomass [35]. This last topic was studied due to the competition for arable land generated between energy-oriented crops versus traditional crops.

A third group of highly-cited articles is related to the characteristics of sugarcane bagasse for production of chemical groups that can be chemically modified to produce adsorbent materials with new properties [36], and another line of research is related to the genomics of the virus that attack sugarcane (sugarcane streak virus) [37]. In general, the most-cited articles are related to alternative uses for sugarcane.

### 3.3. Science Mapping

**Figure 3:**
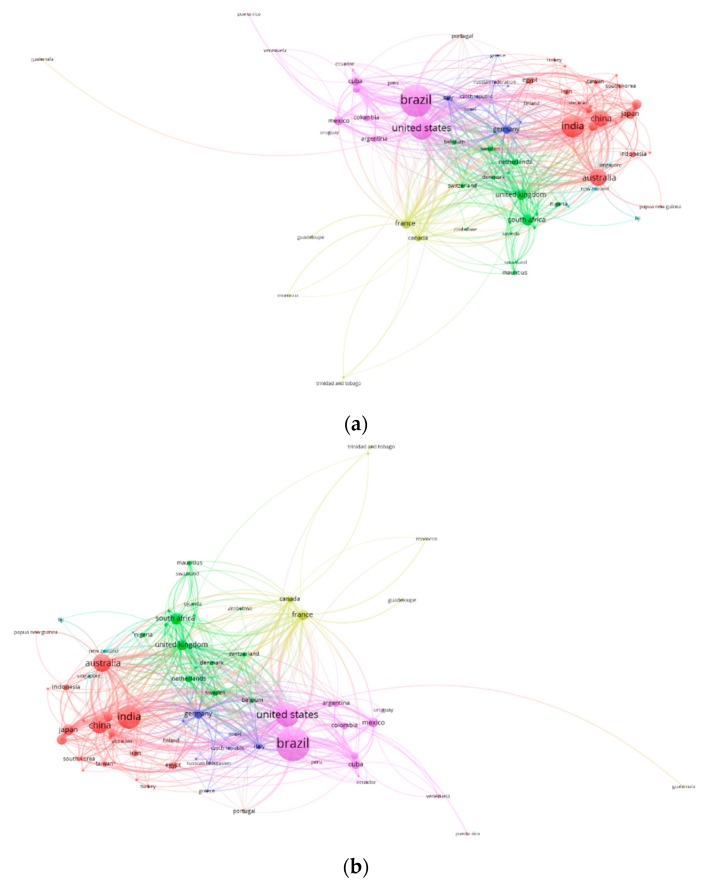
International country co-authorship network of publications related to sugarcane research. (**a**) By number of documents. (**b**) By number of citations.

Science maps are used in order to visualize the relationship between related items. Distance-based maps are maps in which distance reflects relationships, i.e., smaller distance reflects a stronger relationship [14]. In Figure 3, we present a co-authorship country network using VOSviewer for total documents published and citations. A node represents a country and its size indicates its contribution to the research on sugarcane topics. The thickness of the lines reflects the tightness of cooperation between countries. Researchers from a total of 609 countries had publications. A rule of 25 documents per country was used in order to create the map, so a total of 74 countries were retained and 8 clusters were created. In Figure 3a, it can be seen that the countries with the largest number of documents were Brazil (8431), United States (4174), India (4137), Australia (2455), and China (2086). Figure 3b shows the countries with the highest number of citations: Brazil (115,078), United States (89,683), India (44,806), Australia (38,267), and China (25,915). The United States was the country with the most collaboration around the globe. Brazil had the highest level of collaboration with other Latin American countries such as Mexico, Cuba, and Colombia. China, Australia, and other Asian countries showed a strong collaboration network, and India showed collaboration with countries in Asia. France was linked to Morocco and other former French colonies, while other European countries collaborated mainly with African countries.

**Figure 4:**
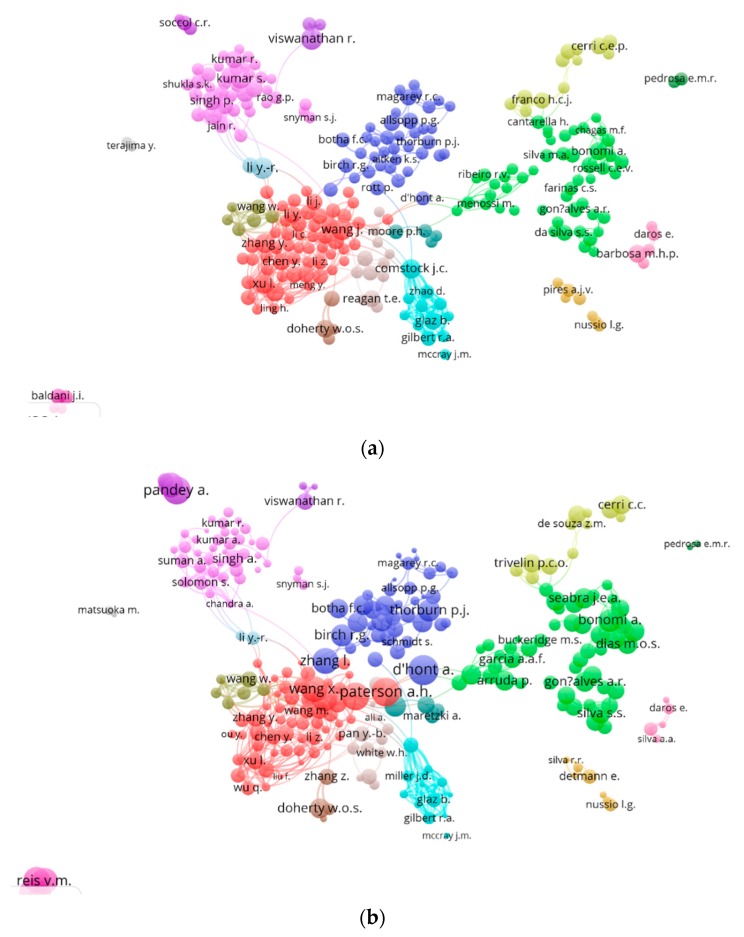
International co-authorship network of publications related to sugarcane research. (**a**) By number of documents. (**b**) By number of citations.

A second pair of maps was created for co-authorship using VOSviewer software. In Figure 4, a node represents an author, and the size represents productivity. We set the threshold at 25 documents and 10 citations per author. The VOSviewer software divided these 264 items into 18 clusters. One color represents one cluster. The author with the most documents was Viswanathan, who works with sugarcane diseases, particularly viruses (109 publications with a total of 963 citations in the sugarcane database used); the second author was Li Y.-R. (106 publications with a total of 619 citations), who publishes research on diverse topics (Figure 3a). The most-cited authors were D’Hont A. (49 documents and a total of 3059 citations in the sugarcane database used), Paterson, A. H. (31 documents and a total of 2736 citations), and Pandey, A. (42 documents and a total of 2619 citations); the research of the first two authors is principally in the area of sugarcane genomics while the last author studies biotechnology. In terms of the clusters, it is clear that the researchers tend to group by country, as collaborations are less limited by geographic distance and language issues; this allows one to observe highly productive researchers in different countries, e.g., Viswanathan in India, Bonomi, A. in Brazil, or Allsopp, P.G. in Australia. The largest cluster is made up of Chinese researchers, a second cluster comprises Brazilian researchers, the third cluster is constituted by Indian researchers, the fourth by Australian researchers, while the rest were clusters with twelve researchers or fewer who do not collaborate to a large extent with other researchers; these are the small independent dots in Figure 4.

**Figure 5:**
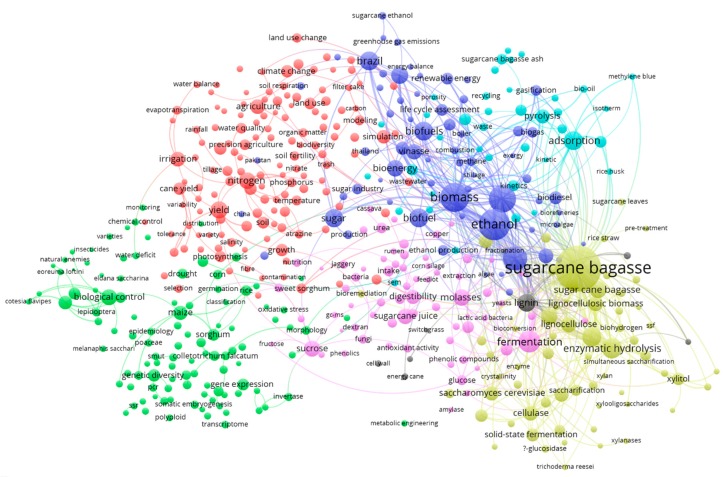
Network showing the co-occurrence of terms in sugarcane research with five co-occurrences.

The map in Figure 5 used as a rule the co-occurrence of at least five times each term, including 500 terms organized into seven clusters. The terms *sugar cane*, *sugarcane*, and *Saccharum* were excluded. The first cluster in the first map included terms related to crop yield (red), the second terms related to genomics (green), the third terms related to sugarcane juice (pink), the fourth bioenergy (bio-ethanol, biofuel, biogas, biomass, etc.) (purple), the fifth included terms related to sugarcane bagasse (yellow), the sixth to decomposition of sugarcane bagasse (light blue), and the seventh to lignin (gray).

## 4. Discussion

The research regarding sugarcane has mainly focused on sugarcane bagasse, especially due to its use as biomass for ethanol or biofuel production. While the second most common line of research has used a more agronomic approach regarding the increase of sugarcane yields. A brief discussion of the contributions regarding these main topics follows.

### 4.1. Sugarcane Bagasse

Sugarcane bagasse is a complex material that is the major by-product of the sugarcane industry. It was used mainly by the sugar mills as fuel for boilers [40], and nowadays it is also used for ethanol and biogas production [41] as well as for electricity production through cogeneration [42] and other commercial applications in other sectors. One of the main applications of bagasse is the bioconversion process that makes it an adequate fermentation media for microorganism production [43]. Another important research area regarding sugarcane bagasse is related to its use as a solid fuel for energy generation and as raw material for production of liquid fuels and chemicals [44]; therefore, a significant amount of research has been done in order to evaluate different pretreatments to improve its energy production capacity [45], e.g., enzyme addition and solids loading [46]. A third venue of research includes other uses of sugarcane bagasse for other industries, e.g., the textile [47], plastic [48], construction [49,50], pharmaceutical [51], and chemical industries [52], among others. Some of these alternative uses have greater added value than the current and conventional ones [53]. Finally, an important research area focuses on evaluating sugarcane bagasse for animal feed production [54].

### 4.2. Ethanol, Biomass, Biofuel, and Bioenergy

Alternative renewable sources of energy have been used in various countries, and biomass such as cellulose from agroindustrial waste is the most abundant biomass in the world; it has been considered a renewable, inexpensive, cost effective, and sustainable source for commercial production of bio-energy as bio-ethanol [55]. Other authors argue that ethanol has significantly grown in popularity due to government regulations and economic incentives [56], but that this kind of feedstock is essentially food, and other sources for bio-ethanol production exist that could substitute sugarcane [57]. At the same time, the demand for sugarcane used as biofuel in countries such as Brazil [58] has led to an increase in the sugarcane production area, in some cases, converting pasturelands to sugarcane fields [59]. This has been an important debate among researchers, generating many publications oriented towards the demonstration of its technical and economic viability for promising new raw materials, e.g., microalgae [60] or alternative energy sources, as well as the way to process them and the technology developed to that end, representing a threat for sugarcane based energy production. In addition, this has been an important debate for other countries that have followed this line of production, e.g., India [61], the Philippines [62], Nigeria [63], Mexico [64], and Thailand [65].

Another research area is related to second generation bioethanol, which is produced from lignocellulosic materials, in particular from sugarcane trash. Unlike sugarcane bagasse, sugarcane trash is previously burned in order to improve the harvest procedure and it is normally left in the field for agricultural purposes [66]; therefore its use for bioenergy requires the use of hydrolysis. It differs from first generation ethanol, requiring a pre-treatment and hydrolysis to break the fibrous material and enable its use [67]. The technologies for second and third generation ethanol production, which uses algae as raw material [68], are expensive and not economically viable [69], yet they have become an important research venue.

Sustainability has been an important research topic for sugar cane [70], approached from diverse angles such as CO_2_ emissions reduction through electricity cogeneration from sugarcane bagasse [71], environmental impact assessment [3], social dimension analysis [72], corporate social responsibility [71], and Life Cycle Sustainability Assessment (LCSA) [73].

### 4.3. Yield

Another important research area for sugarcane corresponds to the field of agronomy. As the major objective of crop production is to increase yields, researchers have focused on diverse topics such as combating pests and diseases. In the case of pests, the main pests studied have been the sugarcane borer [74,75], termites [76], and rodents [77], while the main diseases studied have been: mosaic infection [78], eye leaf spot [79], and red rot [80]. For both pests and diseases, chemical [81] and biological [82,83] control have been evaluated.

Sugarcane breeding has been an important area for yield increase, as more resistant cultivars have been developed, i.e., cultivars tolerant to chilling stress [84], drought stress [85], or pest resistant cultivars [86]. Another important advance is the hybridization of sugarcane with other species in order to improve cultivated sugarcane, especially in order to facilitate their use in biorefinery [87], such as *Erianthus arundinaceus* [88].

There have been some major advances in terms of analyzing the sugarcane genome, which will allow future genomic assisted breeding programs not only for increasing sugar production [89], or more resistant plants under various types of stress [90], but also for obtaining varieties with a more efficient conversion of sugarcane biomass into fermentable sugars for biofuel production [91]. The use of biotechnology has also been important in establishing the performance of micropropagated plants [92], for developing varieties that are tolerant to salt and drought [93], or genetically modified cultivars [94]. The evaluation of fertilizers [95,96], herbicides [97], soil conservation [98], and irrigation system efficiency [99] have also been important topics, as well as the use of various agricultural techniques to improve yields, such as precision agriculture [100] and remote sensing [101]. Sustainability has been also a significant research topic, for example: minimum tillage systems in sugarcane [102].

## 5. Conclusions

We have presented a bibliometric mapping analysis of the research on sugarcane, using data from titles, abstracts, and keywords of articles published in leading journals and other peer-reviewed documents available in the SCOPUS database from 1858 to 2019, and this was subsequently analyzed with the software VOSviewer. A performance analysis was carried out in order to analyze the most relevant journals, countries, and institutes publishing topics related to sugarcane, and a citation analysis and science mapping were also carried out. The two most important countries publishing research regarding sugarcane were Brazil and the United States, they were also the most cited. The most prolific authors tend to publish on diverse topics regarding sugarcane, and most of them tend to rely heavily on their national collaboration network. The analysis of the co-occurrence of terms led us to observe that the main research areas were sugarcane bagasse and terms related to bioenergy and alternative uses, and on a second level of relevance agronomy topics related to increasing crop yields.

Bibliometric mapping allows researchers to understand the evolution of the knowledge of the field in which they are active, providing them with a critical vision of what they are doing and where they should aim to go. We do not pretend to offer a unique vision of the field; we understand that different experts would even offer different interpretations of the results we have presented, yet we consider that this first attempt to visualize the abundance of publications regarding sugarcane in their totality is in itself a good starting point for further scientific discussion.

The limitation of the study is that it relies exclusively on articles published in SCOPUS database, which might not be sufficient to represent all of the sugarcane literature, especially articles in the Google Scholar database or other major publications such as those of the ISSCT and the IAPSIT. Authors that ranked highly in our database might not correspond with the Google Scholar information; therefore, our results may not reflect the real impact of some researchers, but they do provide a general overview of research in the sugarcane field. Due to the lack of previous research, we decided to use a broader approach including all published articles that might contain the term sugarcane/sugar cane, therefore, many publications that only use sugarcane as a reference appeared as most cited. A more refined study is recommended.

## Figures and Tables

**Table 1:** Journals, institutes, and countries with published research on sugarcane.

Rank	Journal	TPs	Country/Region	TPs	Institute	TPs
1	Sugar Tech	892	Brazil	8444	Universidade de Sao Paulo—USP	2420
2	International Sugar Journal	650	United States	4189	UNESP-Universidade Estadual Paulista	1393
3	Bioresource Technology	553	India	4113	Universidade Estadual de Campinas	1119
4	Revista Brasileira de Zootecnia	246	Australia	2458	Sugar Research Australia	665
5	Biomass and Bioenergy	212	China	2086	Empresa Brasileira de Pesquisa Agropecuaria—Embrapa	530
6	Industrial Crops and Products	199	South Africa	923	University of Florida	500
7	Pesquisa Agropecuaria Brasileira	168	United Kingdom	895	USDA Agricultural Research Service, Washington DC	482
8	Plos One	157	Japan	853	Universidade Federal de Sao Carlos	474
9	Applied Biochemistry and Biotechnology	151	France	782	Universidade Federal de Vicosa	468
10	Cuban Journal of Agricultural Science	148	Mexico	737	University of Queensland	438

TPs: Total Publications. Source: SCOPUS (28 August 2019).

**Table 2:** The top 10 highly-cited papers related to sugarcane research (1858–2019 (27 August)).

Rank	Authors (Year)	Title	Source Title	Cited by
1	Brennan and Owende [30] (2010)	Biofuels from microalgae-A review of technologies for production, processing, and extractions of biofuels and co-products	Renewable and Sustainable Energy Reviews	2271
2	Paterson et al. [32] (2009)	The Sorghum bicolor genome and the diversification of grasses	Nature	1657
3	Chisti [31] (2008)	Biodiesel from microalgae beats bioethanol	Trends in Biotechnology	1260
4	Saha [34] (2003)	Hemicellulose bioconversion	Journal of Industrial Microbiology and Biotechnology	1200
5	Kim and Dale [38] (2004)	Global potential bioethanol production from wasted crops and crop residues	Biomass and Bioenergy	1144
6	Wan Ngah and Hanafiah [36] (2008)	Removal of heavy metal ions from wastewater by chemically modified plant wastes as adsorbents: A review	Bioresource Technology	1116
7	Sánchez and Cardona [33] (2008)	Trends in biotechnological production of fuel ethanol from different feedstocks	Bioresource Technology	1068
8	Waterfield et al. [37] (1983)	Platelet-derived growth factor is structurally related to the putative transforming protein p28sis of simian sarcoma virus	Nature	994
9	Balat and Balat [39] (2009)	Recent trends in global production and utilization of bio-ethanol fuel	Applied Energy	830
10	Sims, Mabee, Saddler and Taylor [35] (2010)	An overview of second generation biofuel technologies	Bioresource Technology	808

Source: SCOPUS (27 August 2019).
